# Does silvoagropecuary landscape fragmentation affect the genetic diversity of the sigmodontine rodent *Oligoryzomys longicaudatus*?

**DOI:** 10.7717/peerj.3842

**Published:** 2017-09-29

**Authors:** Daniela Lazo-Cancino, Selim S. Musleh, Cristian E. Hernandez, Eduardo Palma, Enrique Rodriguez-Serrano

**Affiliations:** 1 Departamento de Zoologia, Universidad de Concepción, Concepción, Biobío, Chile; 2 Departamento de Oceanografía, Universidad de Concepción, Concepción, Biobío, Chile; 3 Departamento de Ecología, Pontificia Universidad Católica de Chile, Santiago, Chile

**Keywords:** Conservation genetics, Sigmodontinae, Genetic diversity, Genetic structure, Least cost path, Surrounding matrix

## Abstract

**Background:**

Fragmentation of native forests is a highly visible result of human land-use throughout the world. In this study, we evaluated the effects of landscape fragmentation and matrix features on the genetic diversity and structure of *Oligoryzomys longicaudatus,* the natural reservoir of Hantavirus in southern South America. We focused our work in the Valdivian Rainforest where human activities have produced strong change of natural habitats, with an important number of human cases of Hantavirus.

**Methods:**

We sampled specimens of *O. longicaudatus* from five native forest patches surrounded by silvoagropecuary matrix from Panguipulli, Los Rios Region, Chile. Using the hypervariable domain I (mtDNA), we characterized the genetic diversity and evaluated the effect of fragmentation and landscape matrix on the genetic structure of *O. longicaudatus*. For the latter, we used three approaches: (i) Isolation by Distance (IBD) as null model, (ii) Least-cost Path (LCP) where genetic distances between patch pairs increase with cost-weighted distances, and (iii) Isolation by Resistance (IBR) where the resistance distance is the average number of steps that is needed to commute between the patches during a random walk.

**Results:**

We found low values of nucleotide diversity (*π*) for the five patches surveyed, ranging from 0.012 to 0.015, revealing that the 73 sampled specimens of this study belong to two populations but with low values of genetic distance (*γ*_*ST*_) ranging from 0.022 to 0.099. Likewise, we found that there are no significant associations between genetic distance and geographic distance for IBD and IBR. However, we found for the LCP approach, a significant positive relationship (*r* = 0.737, *p* = 0.05), with shortest least-cost paths traced through native forest and arborescent shrublands.

**Discussion:**

In this work we found that, at this reduced geographical scale*, Oligoryzomys longicaudatus* shows genetic signs of fragmentation. In addition, we found that connectivity between full growth native forest remnants is mediated by the presence of dense shrublands and native forest corridors. In this sense, our results are important because they show how native forest patches and associated routes act as source of vector species in silvoagropecuary landscape, increasing the infection risk on human population. This study is the first approach to understand the epidemiological spatial context of silvoagropecuary risk of Hantavirus emergence. Further studies are needed to elucidate the effects of landscape fragmentation in order to generate new predictive models based on vector intrinsic attributes and landscape features.

## Introduction

Habitat fragmentation is widely recognized as a major threat to global biodiversity (Brooks et al., 2002; Secretariat of the Convention on Biological Diversity, 2005). In this process, a large wild habitat changes into a number of small isolated patches as consequence of human activities (Wilcove, McLellan & Dobson, 1986; Fahrig, 1997; Fahrig, 2003). Those changes imply gradual or accelerated reduction of original habitat’s area (Young, Boyle & Brown, 1996; Lande, 1998; Fahrig, 2003). The main intraspecific consequences of habitat fragmentation are discontinuities on the distribution of resources and species’ optimal environmental conditions, leading to a decrease in connectivity among fragmented populations (Vos & Stumpel, 1995; Lees & Peres, 2008). Thus, fragmentation isolates population, reduces gene flow, the genetic diversity and the effective population size, favoring genetic processes such as drift and inbreeding (Allendorf & Luikart, 2007; Johansson, Primmer & Merilä, 2007; Taylor et al., 2011). In addition, habitat fragmentation can affect the fitness reducing adaptive responses to local selective environments and may cause local extinction (Bolger et al., 1997; Frankham, 2005; Johansson, Primmer & Merilä, 2007; Willi et al., 2007; Bijlsma & Loeschcke, 2012).

Recently, it has been proposed that the structure of the landscape matrix is the main modulator of the consequences of fragmentation on biodiversity (Gascon et al., 1999; Debinski, 2006). Accordingly, if the surrounding matrix has structural similarity with the original habitat remnants, the inter-patch migration is granted avoiding important reduction of patch species richness (Gascon et al., 1999; Ricketts, 2001; Prugh et al., 2008; Franklin & Lindenmayer, 2009; Driscoll et al., 2013). Although this proposal has strong support on different fragmented systems, the genetic consequence of the fragmentation in vertebrates has a strong bias to Tropical Forest species (Radespiel & Bruford, 2014). Further, the intraspecific consequence of the matrix permeability has been studied mainly on European vertebrate or insect species (e.g., McRae, 2006; Arens et al., 2007; Emaresi et al., 2011; Van Strien, Keller & Holderegger, 2012).

The patch’s connectivity is often measured through fixation indexes and isolation by distance (Manel et al., 2003). However, Arens et al. (2007) use an explicit calculation of matrix permeability variables for the study of connectivity among Moor frog patches from Netherlands, highlighting the importance of incorporating the landscape complexity on the evaluation of genetic connectivity. This implies that a simple isolation by distance model is insufficient to explain the genetic diversity in a system of patches surrounded by a matrix of several land-uses. Thus, numerous approaches have been developed in recent years, accounting for the matrix complexity with different theoretical foundations. According to Van Strien, Keller & Holderegger (2012), these approaches can be categorized into two groups: those using transects and those using matrix features to establish landscape cost/resistance surfaces. For the latter, there are two popular approaches, least-cost distance model and the circuit theory model (e.g., Walker & Craighead, 1997; Adriaensen et al., 2003; McRae, 2006). The least-cost distance models minimize the travel distance among habitat patches and the cost traversed, offering the shortest cumulative cost-weighted distance (optimal route) between an origin patch to a destination patch. On the other hand, McRae (2006) proposed a model based on circuit theory that “predicts a positive relationship between genetic differentiation and the resistance distance, a distance metric that exploits precise relationships between random walk times and effective resistances in electronic networks”. Thus, these new approaches allow to understand the effecst on the genetic diversity of the patch-matrix dynamics.

During the last 30 years, the Mediterranean and the Temperate Chilean landscapes have been strongly modified by silvoagropecuary activities (agricultural, lumbering and industrial forestry activities), where remnants of native forests are restricted to zones with difficult access (Bustamante & Grez, 1995; Aguayo et al., 2009). Then, these patches of natural habitats constitute a highly fragmented environment, where patches are surrounded by different productive crops, exotic forestry species (mainly *Eucalyptus globulus* and *Pinus radiata*), and secondary regrowth native forest.

A frequent species in the Mediterranean and Temperate Forests of Chile is the long-tailed pygmy rice rat (“Colilargo”), *Oligoryzomys longicaudatus* (Bennett, 1832). This sigmodontine rodent has a broad distribution in Chile and Argentina. In Chile it occurs from 27°S to 54°S (Belmar-Lucero et al., 2009), whereas in Argentina ranges from 36°S to 51°S on the eastern slope of the Andes mountains (Carbajo & Pardiñas, 2007). *Oligoryzomys longicaudatus* shows a high vagility and a large home range 320–4,800 m^2^, with seasonal fluctuations (Murúa, González & Meserve, 1986). The species is mainly granivorous, inhabiting microhabitats with dense foliage, which could be related to its saltorial mode of locomotion and as a mechanism to avoid predators (Murúa, González & Jofre, 1980; Murúa & González, 1982). Molecular studies based on cytochrome *b* mitochondrial DNA (mtDNA) sequences have shown a marked genetic homogeneity along the species geographic distribution (Palma et al., 2005). However, studies based on hypervariable domain I (HVI) of the mtDNA recovered a geographical structure of populations in agreement with the ecoregions and the three recognized subspecies along the species range (Palma et al., 2012a), and a temporal genetic variability at local scale (Boric-Bargetto et al., 2012). In addition, *O. longicaudatus* has been the focus of numerous epidemiological studies, given that the species is the major reservoir of the Andes strain of Hantavirus that causes a cardiopulmonary syndrome to human populations with a mortality rate of about 35% (Toro et al., 1998; Martinez-Valdebenito et al., 2014). Thus, to evaluate the effects of changing landscapes on the migration and connectivity of *O. longicaudatus* populations*,* and the potential effect on infection rates, especially on peri-urban areas, constitute highly relevant issues to the ecology, the genetics and the epidemiology of this species (Torres-Pérez et al., 2004). Therefore, in this study we evaluated the effects of landscape fragmentation and matrix structure on the genetic diversity and genetic structure of *O. longicaudatus.* We focused our work on a portion of the southern Temperate Forest (the Valdivian Rain Forest) where human activities have produced a strong impact on natural habitats, and where an important number of human cases of Hantavirus have been reported in Chile (http://epi.minsal.cl/hantavirus-materiales-relacionados/).

## Materials & Methods

### Study site and specimens analyzed

The Valdivian Temperate Forest—southern Chile and nearby Argentina—is one of the 25 biodiversity hotspots of the world threatened by anthropogenic activities (Olson, 1998; Myers et al., 2000). This biome/ecoregion has been considered a biogeographic island that harbors a quite diverse assemblage of mammals where native species are mostly restricted to national parks and rural areas with fragmented landscapes (Echeverría et al., 2007). For this study, we took samples of *O. longicaudatus* from five different patches of dense native, full growth, temperate forests in a total area of 3.5 km^2^. The specimens were trapped with standard Sherman traps (8 × 9 × 23 cm; H. B. Sherman Traps, Inc., Tallahassee, FL, USA). The field trapping procedure was conducted through a regular grid sampling design, setting 450 traps night for three nights, and we used oat and vanilla as bait.

**Figure 1:**
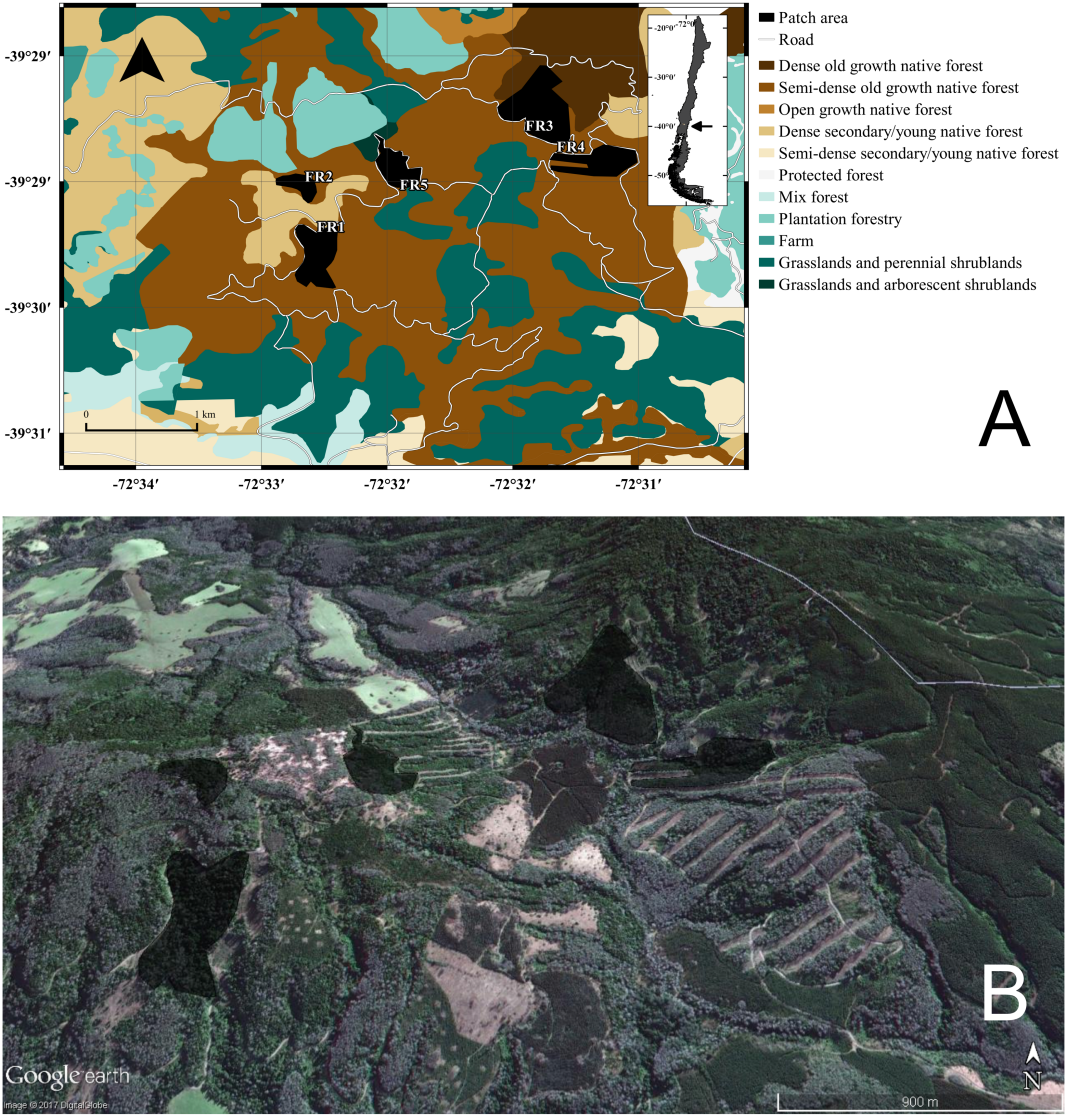
GIS representation and satellite image of the study site. (A) Land uses, roads, and native forest patches (fragments “FR*X”*) where individuals of *Oligoryzomys longicaudatus* were sampled. (B) Google Earth image of the study site depicting the fragments and the high heterogeneity of the land uses. Map Data: Google Earth, DigitalGlobe.

The study was conducted during the autumn and winter of 2007 in the locality of Curirruca, Panguipulli province, Los Rios region southern Chile (39°48′30″S, 73°14′30″W; Fig. 1). The captures were conducted under the Chilean Government authorization: Resolución Exenta No 7325 (December 30, 2005; from Servicio Agrícola y Ganadero, Ministerio de Agricultura, Gobierno de Chile). We selected this temporal window because it did not match with the reproductive period of the species and the mobility among patches should be reduced (Murúa, González & Meserve, 1986). The patches sampled were surrounded by a matrix—the rest of landscape after exclusion of habitat patches—characterized by recent adult and/or harvested plantations, grasslands and shrublands areas, agricultural fields, and/or adjacent to forest roads (Table S1). The appropriate landscape soil uses for the sampling year were obtained from the Chilean Government National Environmental Information System—SINIA (http://ide.mma.gob.cl/).

The present study was conducted using blood samples and liver tissue from 73 specimens of *O. longicaudatus,* collected in this area (Table S2). All specimens were handled following the standard bioethical and biosafety protocols proposed by the American Society of Mammalogists (ASM; Sikes & Gannon, 2011), and the Center for Diseases Control and Prevention (CDC; Mills et al., 1995), respectively.

### Laboratory methods

DNA was extracted using the Wizard® Genomic DNA Purification Kit (PROMEGA, Madison, WI, USA). Through the polymerase chain reaction (PCR) we amplified ∼1100 bp from mtDNA from which we used 527 bp corresponding to the hypervariable subunit I (HVI) and part of the conservative domain of the Control Region. The mammalian mtDNA hypervariable regions are included within the extended terminal associated sequences (ETAS) and conserved sequence block (CSB; Vigilant et al., 1991; Sbisà et al., 1997; Pesole et al., 1999). The evolution of D-loop region in mammals is characterized by a strong rate heterogeneity among sites, tandem repeated elements and high frequency of insertion/deletion events (Saccone, Pesole & Sbisà, 1991; Wakeley, 1993; Sbisà et al., 1997; Pesole et al., 1999). The ETAS and CSB domains evolve fast enough to be used for population genetics studies (Wakeley, 1993; Sbisà et al., 1997; Pesole et al., 1999; Rosel et al., 1999; Kerth, Mayer & König, 2000; Matson et al., 2000).

We used primers DLO-L (5′ CGG AGG CCA ACC AGT AGA 3′) and DLO-H (5′ TAA GGC CAG GAC CAA ACC 3′; Belmar-Lucero et al., 2009; Palma et al., 2012a) according to the following thermal profile: an initial denaturation of 5 min at 94 °C, followed by 25 or 30 cycles of denaturation for 30 s at 94 °C, annealing for 30 s at 57 °C and extension for 1 min 30 s at 72 °C, and a final extension of 5 min at 72 °C. The PCR products were sent to Macrogen (http://dna.macrogen.com/) for purification and sequencing (Applied Biosystems 3730XL sequencer; Applied Biosystems, Foster City, CA, USA). Sequences were edited with BioEdit v 7.2.5 (Hall, 1999) and aligned using ClustalW (Thompson, Higgins & Gibson, 1994).

### Data analyses

#### Genetic diversity

To describe the genetic diversity in all the patches studied we used the DnaSP Software v 5.10.01 (Librado & Rozas, 2009) to estimate the number of haplotypes (Nh), segregating sites (S), the haplotype diversity (Hd) and the nucleotide diversity (π). The same software was used to calculate the Gamma_ST_ (γ_ST_; Nei, 1982) a statistical index of genetic differentiation that represents an unbiased estimate of the population subdivision fixation index (*F*_ST_) and its use is more appropriate for haplotype data. Statistical significance of genetic differentiation was tested using Hudson’s nearest neighbor statistics (*S*_nn_) with 1,000 permutations in DnaSP. *S*_nn_ statistics indicates the frequency with which nearest neighbor sequences are found in the same group (Hudson, 2000).

#### Fragmentation effect

To evaluate the fragmentation effects on the genetic structure of *O. longicaudatus* we estimated the number of panmictic units in the landscape surveyed using the package GENELAND v 3.2.2 (Guillot, Mortier & Estoup, 2005) in the R software (R Core Team, 2016). For this, we follow the proposal of Guillot et al. (2012) codding the data in such a way that the various haplotypes of mtDNA are recoded as alleles of a single locus. This package implements a statistical model with Bayesian inference and uses geo-referenced data of the sequenced individuals, inferring and locating genetic discontinuities between populations. The number of clusters was determined by running MCMC (Markov chain Monte Carlo) iterations to estimate *K* (i.e., the most probable number of populations). The analysis was performed in both non-correlated and correlated model, allowing values of *K* to vary from 1 to 5, running MCMC with 10,000,000 iterations sampling each 1,000. To choose the best model that fits the data, a log10 Bayes Factor (BF) with 1,000 bootstrap replicates was performed in Tracer v 1.5 (http://tree.bio.ed.ac.uk/software/tracer/). The model that better fitted our data was the correlated allele frequency model, which assumes that rare alleles in a certain populations are also rare in other populations (Guillot, Mortier & Estoup, 2005).

#### Landscape matrix effect

For the identification of landscape matrix effects on the genetic structure of *O. longicaudatus* we followed three approaches. First, we performed isolation by distance test (IBD) as a null model, because this model contains no information about landscape features, where dispersal occurs in homogenous geographic spaces (Nowakowski et al., 2015). IBD was performed using vegan package 2.4-1 package (Oksanen et al., 2016) implemented in R (R Core Team, 2016). IBD was tested using a mantel test with 119 permutations between a matrix of genetic distances (γ_ST_) and a matrix of geographic distances between the five patches. Second, we used Least-Cost Path (LCP) analysis. In LCP, genetic distances between patch pairs increase with cost-weighted distances, taking into account the friction effects of the landscape on the individual movement process (Adriaensen et al., 2003; Epps et al., 2007). Third, we used isolation by resistance (IBR) analysis, where dispersal occurs in heterogeneous landscapes and the resistance distance is the average number of steps that is needed to commute between the patches during a random walk that is calculated using the circuit theory (McRae, 2006).

To estimate the distances under LCP and IBR models we used the package gdistance (Van Etten, 2017). For this, we first fed the gdistance package with a raster file containing the landscape features of the study area classified in five classes: native forest (all age classes), grassland and shrublands (with/out arborescent elements), farm (agricultural use), plantation forestry (monoculture of exotic species, *eg. Pinus radiata*) and mix forest (zone with both native and introduced trees). The raster resolution was 0.008 × 0.0006 pixels, but for technical feasibility we created a raster layer with larger cells (0.048 × 0.0034 pixels of resolution) using raster package 2.5-8 (Hijmans, 2015; Hijmans, 2016) in R (R Core Team, 2016). Second, we used transition function of gdistance to create a transitions matrix which represents the transition from one cell to another on a grid where each cell is connected to its 8 neightbours. In short, this function calculates the conductance values from the values of each pair of cells to be connected (Van Etten, 2017). However, there are two distance distortions that need to be corrected; diagonal neighbors are more remote from each other than orthogonal neighbours, and on a longitude-latitude grids, West–East connections are longer at the equator and shorter towards the poles. To solve these distortions, we used the geoCorrection function. For the transition matrix used in LCP, this function divides each value from the matrix by distance between cell centers (Van Etten, 2017). On the other hand, for the transition matrix used in IBR, the function weights the probability of reaching an adjacent cell in a random walk by making it proportional to the surface covered by the cell, multiplying the North–South transition values with the cosine of the average latitude of the two cell centers that are being connected (Van Etten, 2017). To calculate least-cost distances between patches, we used costDistance function that computes the cost units as the reciprocal of the values in the transition matrix using the Djkstra’s algorithm (Dijkstra, 1959). To calculate the resistance distances between patches, we used commuteDistance function, this function uses the algorithm implemented by Fouss et al. (2007) to calculate the expected random walk commute time between patches, resulting in the average number of steps needed to commute between the locations (Van Etten, 2017).

To perform the correlation between genetic distances (γ_ST_) with both, LCP and IBR distances, we used vegan package 2.4-1 package (Oksanen et al., 2016) in R (R Core Team, 2016) performing a mantel test with 119 permutations. This number of permutations is the maximum number allowed to avoid duplication due the size of matrix distance (i.e., 5 × 5). Finally, we used gdistance 1.1-9 package (Van Etten, 2015) to trace the quickest path among pairs of patches for LCP model applying shortestPath function which calculates the shortest path from one patch to another. This allowed us to know if there were specific types of soil use (the surrounding matrix) that the “Colilargo” uses as a corridor.

## Results

### Genetic diversity

We found a total of 43 segregating sites and 36 haplotypes out from 73 sequences (Table 1). All patches showed a high haplotype diversity (Hd), although FR5 showed the lowest value (0.867) whereas FR2 exhibited the highest values (0.987) (Table 1). Regarding nucleotide diversity (π), it was low, ranging from 0.012 (FR4) to 0.015 (FR1) (Table 1).

**Table 1:** Descriptive statistics of the genetic variation of *O. longicaudatus* sequences for each sampled patch.

Patches	N	S	Nh	Hd ± SD	π± SD
FR1	12	22	9	0.939 ± 0.058	0.015 ± 0.002
FR2	18	33	16	0.987 ± 0.023	0.014 ± 0.001
FR3	9	20	8	0.972 ± 0.064	0.013 ± 0.002
FR4	24	28	13	0.880 ± 0.056	0.012 ± 0.002
FR5	10	21	7	0.867 ± 0.107	0.013 ± 0.002
Total	73	43	36	0.954 ± 0.012	0.014 ± 0.001

**Notes.**

N Number of individuals S segregating sites Nh haplotype number Hd haplotype diversity π nucleotide diversity SD standard deviation

### Genetic structure

**Figure 2:**
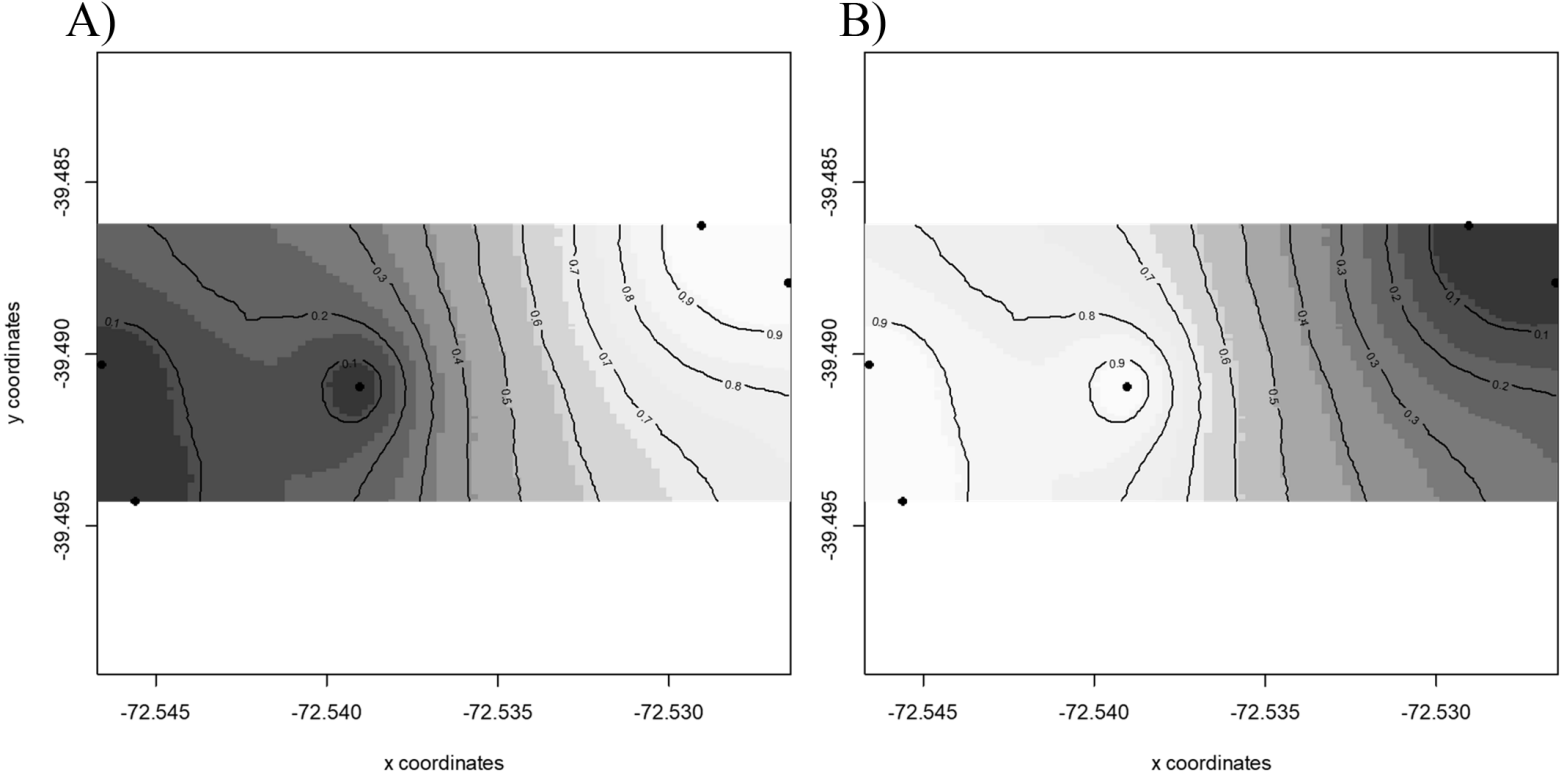
Spatial population structure. GENELAND analyses with posterior probability isoclines denoting the extent of genetic landscapes. Black dots represent patches analyzed. White indicates regions with the greatest posterior probability of inclusion, whereas diminishing probabilities of inclusion are proportional to the degree of coloring. (A) Map of posterior probability to belong to cluster 1; (B) map of posterior probability to belong to cluster 2.

Results of GENELAND v 3.2.2 (Fig. 2) for population genetic structure inferred that the most probable number of clusters of individuals was two (*K* = 2). The first cluster grouped sequences from FR3 and FR4 (Fig. 2A). The second cluster joined the patches FR1, FR2 and FR5 (Fig. 2B). We found high posterior probabilities (0.9) for cluster assignation (Fig. 2).

Pairwise γ_ST_ values (Table 2) revealed the occurrence of genetic differentiation recorded for FR3 and FR5 (γ_ST_ = 0.099, *p* = 0.046), and between FR4 and FR5 (γ_ST_ = 0.085, *p* = 0.044). The remaining patches showed γ_ST_ values ranging from 0.022 to 0.096 (not significantly different from 0).

### Landscape matrix effects

**Figure 3:**
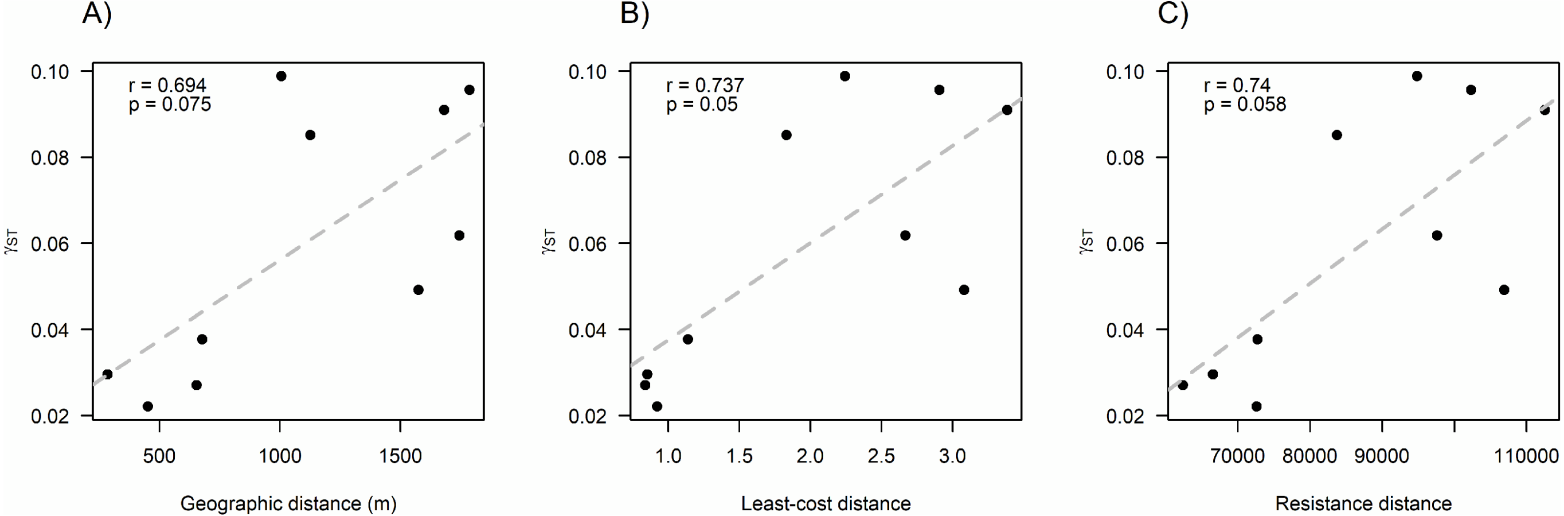
Effect of fragmentation and landscape matrix on the genetic structure of *O. longicaudatus*. Graphics of Pearson correlation coefficient (*r*). (A) Isolation by Distance (IBD), (B) The Least-cost Path (LCP), and (C) Isolation by Resistance (IBR). *r* value corresponds to Pearson correlation coefficient and *p* values correspond to significance (*p* ≤ 0.05).

**Figure 4:**
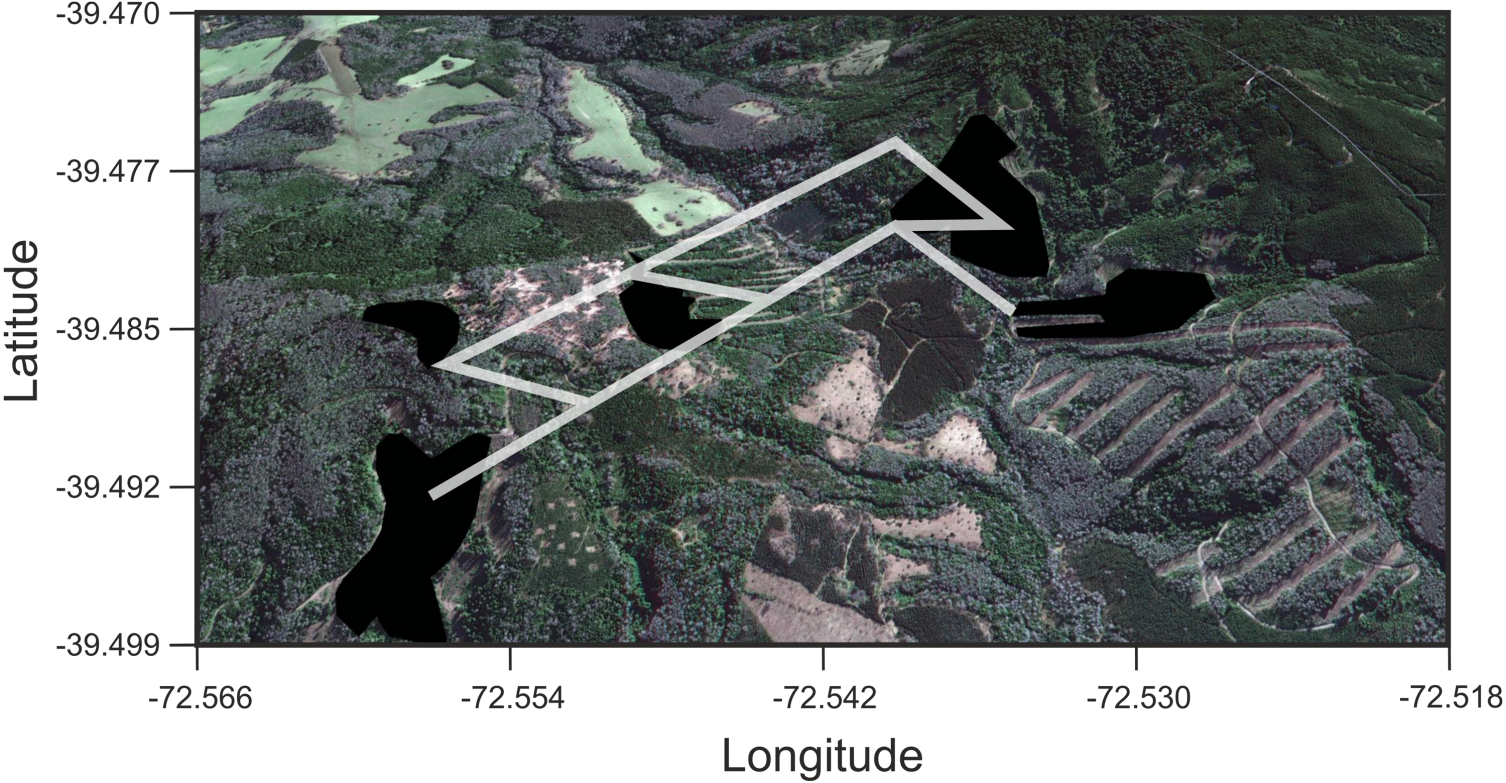
Results of Least Cost Paths analyses. Satellite image of the study site depicting the fragments surveyed and the least cost paths inferred for *Oligoryzomys longicaudatus* in the highly fragmented Valdivian Rainforest of southern Chile. Google Earth image © 2017 DigitalGlobe.

We found that *O. longicaudatus* did not exhibit significant isolation-by-distance, γ_ST_ was not correlated with geographic distance (*r* = 0.694, *p* = 0.075, Fig. 3A). However, we found for the least cost path approach a significant positive relationship (*r* = 0.737, *p* = 0.05, Fig. 3B). But, the long-tailed pygmy rice rat did not exhibit a significant isolation by resistance relationship for genetic differentiation (*r* = 0.740, *p* = 0.058, Fig. 3C). The shortest paths traced for LCP shows that individuals of *O. longicaudatus* moved with preference through young and all growth native forest, and grassland with arborescent shrublands to connect patch pairs (Fig. 4).

**Table 2:** Pairwise genetic distances between patches (γst) (below diagonal) and S_NN_ significance *P*-values (above diagonal). Bold numbers represent significant values.

	FR1	FR2	FR3	FR4	FR5
FR1	–	0.835	0.735	0.588	0.31
FR2	0.022	–	0.586	0.351	0.379
FR3	0.091	0.049	–	0.346	0.046
FR4	0.096	0.062	0.030	–	0.044
FR5	0.038	0.027	**0.099**	**0.085**	–

## Discussion

In this work, we found that, at this reduced geographical scale*, Oligoryzomys longicaudatus* shows genetic signs of fragmentation. Also, we found that genetic distance between patches showed best fitting to a LCP model. In addition, we found that connectivity between full growth native forest remnants is mediated by the presence of dense shrublands and native forest corridors. The latter can be composed by different age and health status native formations (Fig. 1B; Fig. 4). These results complement previous efforts to understand the association between landscape attributes and the genetic diversity of this species. Previous studies have been focused on regional and local scales proposing relationships between haplogroups and ecogeographic regions, as well as latitudinal genetic structure in local context (Belmar-Lucero et al., 2009; Palma et al., 2012a; Ortiz et al., 2017). Specifically, Ortiz et al. (2017), studying a fragmented landscape in the Argentinian Patagonia, found that landscape features such as lakes, rivers, roads and urban settlements constrain the movement of *O. longicaudatus*, acting as barriers reducing gene flow. Our results support the latter findings since, even at this small scale, showing abrupt changes in land use being the species strongly affected by the fragmentation of the primary habitat.

The long-tailed pygmy rice rat has a marked foraging behavior characterized by the search of seeds, a highly localized and temporally variable resource (Murúa, González & Meserve, 1986). Previous studies on this species suggested high flexibility in habitat use, characterized by an opportunistic behavior and large home range (Murúa & González, 1986; Murúa, González & Meserve, 1986; Spotorno, Palma & Valladares, 2000). In addition, studies based in the Valdivian Rainforest (dense full growth forest at Villarrica National Park) strongly suggest that migration is the modulator of the diversity and temporal genetic structure of *O. longicaudatus* (Boric-Bargetto et al., 2012). Thus, the landscape matrix effects on the genetic diversity and genetic structure of this species would be largely buffered by its high vagility features particularly in the Valdivian Rainforest which is its primary habitat (Murúa, González & Meserve, 1986). However, we found that the LCP model is the best predictor of the genetic distance for the fragments surveyed. This implies that *O. longicaudatus* minimize the tradeoff between distance travelled and the costs traversed. Graphically, our results showed that the shortest paths among patch pairs are across native forest (the species’ primary habitat), representing the routes of maximum efficiency for landscape connectivity, while the rest of land uses would act, in some extent, as barriers.

Our results should be viewed with caution, in terms of some possible consequences derived from our interpretations. If the most efficient routes connecting patches are through native forest, this does not mean that individuals of *O. longicaudatus* occurs only in native forest. If dispersing individuals follow these optimal routes, they would increase the probability of survival reaching the optimal destination (another patch). This is because, dispersal through optimal routes and their associated habitats increases increases the likelihood of finding resources and evading predation (Walker & Craighead, 1997). In fact, Moreira-Arce et al. (2015) found that *O. longicaudatus* is the preferred prey of the culpeo fox (*Lycalopex culpaeus*) only in monocultures, increasing its predation risk in silvoagropecuary landscapes. However, dispersing individuals may not choose the optimal route, and travel through other types of soil use less effectively, because of connectivity among patches. For instance, due to its opportunistic behavior and according to previous ecological studies settled in coastal ranges of the Valdivian Rainforest, this species could be found on grassland—shrublands when the seed availability increases on this area but not on the native forest (Murúa & González, 1986; Murúa, González & Meserve, 1986). In tree monocultures, this rodent is less abundant, but not absent, than in native forest and exhibits an omnivorous diet, where mainly consumes seeds (e.g., *Pinus radiata* seeds) and fruits, and arthropods and mushrooms in less amount (Muñoz-Pedreros, Murúa & González, 1990; Moreira-Arce et al., 2015). Another important caveat of this work is the molecular marker used to infer the genetic structure and diversity of the Colilargo. We used a very variable fragment from the mtDNA, then our results reflect the matrilineal genetic diversity and least cost paths. The major consequence of this choice, is that just a quarter of the total effective population size is used in this study, so the results could underestimate the genetic diversity of each patch, but not the routes, since the latter are estimated from the properties of the landscape (Van Etten, 2017). Finally, given that our sampling period was during the autumn and winter, our results may reflect connectivity aspects of Colilargo modulated by the features of those seasons. Interestingly, a previous ecological work on *O. longicaudatus,* settled in Temperate Forest, shows that a very marked seasonal reproductive period from October to May, which overlaps with the recruitment period from March to April, is followed by population peaks during autumn-winter (Murúa, González & Meserve, 1986). Therefore, our sampling was carried out post-recruitment, so the results of genetic diversity and structuring are relevant to estimate connectivity in this fragmented system since these patterns are the result of migration processes between patches that have already occurred.

*Oligoryzomys longicaudatus* is recognized as the major reservoir of the Andes strain of Hantavirus (ANDV) in Southern South America (Medina et al., 2009). This virus causes the Hantavirus Cardiopulmonary Syndrome (HCPS) disease (Martinez-Valdebenito et al., 2014). In Valdivia, Chile, Mansilla (2006) found that >50% of HCPS cases were associated to silvoagropecuary landscapes (Holz & Palma, 2012). In addition, the long - tailed pygmy rice rat is one of the most common species in the rodent assemblages, where it could potentially infect other wild rodent species with the ANDV (horizontal transmission to coexisting species; Palma et al., 2012b), thus increasing the risk to humans (Polop et al., 2010; Andreo et al., 2012; Barrera & Murúa, 2016). In this sense, our results are important because they show how native forest patches and associated routes act as source of vector species in silvoagropecuary landscape, highly associated to human activities increasing the infection risk on human population. Further studies are required to elucidate the effects of landscape fragmentation at large scales (i.e., ecogeographic), in order to gain a deeper understanding of the underlying causes of HCPS infection risk in the Valdivian Forest. Finally, a general pattern of the consequences of Temperate Forest fragmentation should be based on an important number of species, and future efforts should point out to other endemic species of this ancient landscape of South America.

##  Supplemental Information

10.7717/peerj.3842/supp-1 Table S1 Criteria for delimitation of patch areas Criteria for delimitation of patch areas. Criteria were designed visualizing the patches in Google Earth Pro v 7.1 (http://www.google.com/earth/) and based on Soil Use Cover data of the Chilean National Environmental Information System (http://ide.mma.gob.cl/).

10.7717/peerj.3842/supp-2 Table S2 Data for each individual used in this work Sampling site, patch name, coordinates of specimens, GenBank accession number and voucher number of each specimen used in this study.

10.7717/peerj.3842/supp-3 Supplemental Information 1 Raster File for landscape features Raster file with landscape soil uses for 2007 at the study site with 0.048 × 0.0034 pixels of resolution.

10.7717/peerj.3842/supp-4 Supplemental Information 2 Gamma_st distances_ Genetic distances between patches.

10.7717/peerj.3842/supp-5 Supplemental Information 3 Georreferences for each patch Georreferences (decimal system) for each patch surveyed.

10.7717/peerj.3842/supp-6 Supplemental Information 4 Code to perform the analyzes used in this study Annotated R code. This should be used in combination with the raw data delivered in supplementary materials: “comb_999.grd.zip”, “gammast.txt”, and “lonlat.txt”.

## Additional Information and Declarations

### DNA Deposition

The following information was supplied regarding the deposition of DNA sequences: The Hipervariable Domain I, of the Control Region (mtDNA), sequences used here are available via GenBank accession numbers KY211763 to KY211766, KY211783 to KY211818 and KY211820 to KY211862.

### Data Availability

The following information was supplied regarding data availability: The raw data has been supplied as Supplemental Files.

## References

[ref-1] Adriaensen F, Chardon JP, Blust G De, Swinnen E, Villalba S, Gulinck H, Matthysen E (2003). The application of “least-cost” modelling as a functional landscape model. Landscape and Urban Planning.

[ref-2] Aguayo M, Pauchard A, Azócar G, Parra O (2009). Cambio del uso del suelo en el centro sur de Chile a fines del siglo XX. Entendiendo la dinámica espacial y temporal del paisaje. Revista Chilena de Historia Natural.

[ref-3] Allendorf FW, Luikart G (2007). Conservation and the genetics of populations. 2007.

[ref-4] Andreo V, Provensal C, Levis S, Pini N, Enría D, Polop J (2012). Summer–autumn distribution and abundance of the hantavirus host, *Oligoryzomys longicaudatus*, in northwestern Chubut, Argentina. Journal of Mammalogy.

[ref-5] Arens P, Vander Sluis T, Van’t Westende WPC, Vosman B, Vos CC, Smulders MJM (2007). Genetic population differentiation and connectivity among fragmented Moor frog (*Rana arvalis*) populations in The Netherlands. Landscape Ecology.

[ref-6] Barrera K, Murúa R (2016). Nuevo desafío en Salud Pública: presencia de reservorios de Hanta, *Oligoryzomys longicaudatus* y *Rattus* spp., en aéreas de borde en praderas del sur de Chile. Sustainability, Agri, Food and Environmental Research.

[ref-7] Belmar-Lucero S, Godoy P, Ferrés M, Vial PA, Palma RE (2009). Range expansion of *Oligoryzomys longicaudatus* (Rodentia, Sigmodontinae) in Patagonian Chile, and first record of Hantavirus in the region. Revista Chilena de Historia Natural.

[ref-8] Bijlsma R, Loeschcke V (2012). Genetic erosion impedes adaptive responses to stressful environments. Evolutionary Applications.

[ref-9] Bolger DT, Alberts AC, Sauvajot RM, Potenza P, Mccalvin C, Tran D, Mazzoni S, Soul ME (1997). Response of rodents to habitat fragmentation in coastal southern California. Ecological Applications.

[ref-10] Boric-Bargetto D, Rodríguez-Serrano E, Hernández CE, Jaksic FM, Palma RE (2012). Temporal variation in genetic diversity during an outbreak of *Oligoryzomys longicaudatus* (Rodentia, Sigmodontinae) in a temperate forest of southern Chile. Biochemical Systematics and Ecology.

[ref-11] Brooks TM, Mittermeier RA, Mittermeier CG, Fonseca GABDA, Rylands AB, Konstant WR, Flick P, Pilgrim J, Oldfield S, Magin G, Hilton-Taylor C (2002). Habitat loss and extinction in the hotspots of biodiversity. Conservation Biology.

[ref-12] Bustamante R, Grez AA (1995). Consecuencias ecológicas de la fragmentación de los bosques nativos. Ambiente y Desarrollo.

[ref-13] Carbajo AE, Pardiñas UFJ (2007). spatial distribution model of a hantavirus reservoir, the long-tailed colilargo (*Oligoryzomys longicaudatus*), in Argentina. Journal of Mammalogy.

[ref-14] Debinski DM (2006). Forest fragmentation and matrix effects: the matrix does matter. Journal of Biogeography.

[ref-15] Dijkstra E (1959). A note on two problems in connexion with graphs. Numerische Mathematik.

[ref-16] Driscoll DA, Banks SC, Barton PS, Lindenmayer DB, Smith AL (2013). Conceptual domain of the matrix in fragmented landscapes. Trends in Ecology & Evolution.

[ref-17] Echeverría C, Newton AC, Lara A, Benayas JMR, Coomes DA (2007). Impacts of forest fragmentation on species composition and forest structure in the temperate landscape of southern Chile. Global Ecology and Biogeography.

[ref-18] Emaresi G, Pellet J, Dubey S, Hirzel AH, Fumagalli L (2011). Landscape genetics of the Alpine newt (*Mesotriton alpestris*) inferred from a strip-based approach. Conservation Genetics.

[ref-19] Epps CW, Wehausen JD, Bleich VC, Torres SG, Brashares JS (2007). Optimizing dispersal and corridor models using landscape. Journal of Applied Ecology.

[ref-20] Fahrig L (1997). Relative effects of habitat loss and fragmentation on population extinction. Journal of Wildlife Management.

[ref-21] Fahrig L (2003). Effects of habitat fragmentation on biodiversity. Annual Review of Ecology and Systematics.

[ref-22] Fouss F, Pirotte A, Renders JM, Saerens M (2007). Random-walk computation of similarities between nodes of a graph with application to collaborative recommendation. IEEE Transactions on Knowledge and Data Engineering.

[ref-23] Frankham R (2005). Genetics and extinction. Biological Conservation.

[ref-24] Franklin JF, Lindenmayer DB (2009). Importance of matrix habitats in maintaining biological diversity. Proceedings of the National Academy of Sciences of the United States of America.

[ref-25] Gascon C, Lovejoy TE, Bierregaard Jr RO, Malcolm JR, Stou PC, Vasconcelos HL, Laurance WF, Zimmerman B, Tocher M, Borges S (1999). Matrix habitat and species richness in tropical forest remnants. Biological Conservation.

[ref-26] Guillot G, Mortier F, Estoup A (2005). Geneland: a computer package for landscape genetics. Molecular Ecology Notes.

[ref-27] Guillot G, Renaud S, Ledevin R, Michaux J, Claude J (2012). A unifying model for the analysis of phenotypic, genetic and geographic data. Systematic Biology.

[ref-28] Hall TA (1999). BioEdit: a user-friendly biological sequence alignment editor and analysis program for Windows 95/98/NT. Nucleic Acids Symposium Series.

[ref-29] Hijmans RJ (2015). http://cran.r-project.org/package=raster.

[ref-30] Hijmans RJ (2016). https://cran.r-project.org/web/packages/raster/vignettes/Raster.pdf.

[ref-31] Holz A, Palma RE (2012). Floraciones de bambúes en Chile y Argentina: actual floración masiva del coligue, historia natural y riesgos asociados. Revista Bosque Nativo.

[ref-32] Hudson RR (2000). A new statistic for detecting genetic differentiation. Genetics.

[ref-33] Johansson M, Primmer CR, Merilä J (2007). Does habitat fragmentation reduce fitness and adaptability? A case study of the common frog (*Rana temporaria*). Molecular Ecology.

[ref-34] Kerth G, Mayer F, König B (2000). Mitochondrial DNA (mtDNA) reveals that female Bechstein’s bats live in closed societies. Molecular Ecology.

[ref-35] Lande R (1998). Anthropogenic, ecological and genetic factors in extinction and conservation anthropogenic factors ecological factors environmental fluctuations and catastrophes. Researches on Population Ecology.

[ref-36] Lees AC, Peres CA (2008). Conservation value of remnant riparian forest corridors of varying quality for amazonian birds and mammals. Conservation Biology.

[ref-37] Librado P, Rozas J (2009). DnaSP v5: a software for comprehensive analysis of DNA polymorphism data. Bioinformatics.

[ref-38] Manel S, Schwartz MK, Luikart G, Taberlet P (2003). Landscape genetics: combining landscape ecology and population genetics. Trends in Ecology & Evolution.

[ref-39] Mansilla R (2006). Estudio epidemiológico de una serie de casos de infección por hantavirus y su relación con variables temporo-espaciales en la provincia de Valdivia. Tesis de magíster.

[ref-40] Martinez-Valdebenito C, Calvo M, Vial C, Mansilla R, Marco C, Palma RE, Vial PA, Valdivieso F, Mertz G, Ferrés M (2014). Person-to-person household and nosocomial transmission of Andes, Southern Chile, 2011. Emerging Infectious Diseases.

[ref-41] Matson CW, Rodgers BE, Chesser RK, Baker RJ (2000). Genetic diversity of *Clethrionomys glareolus* populations from highly contaminated sites in the Chornobyl Region, Ukraine. Environmental Toxicology and Chemistry.

[ref-42] McRae BH (2006). Isolation by resistance. Evolution.

[ref-43] Medina RA, Torres-Pérez F, Galeno H, Navarrete M, Vial PA, Palma RE, Ferres M, Cook JA, Hjelle B (2009). Ecology, genetic diversity, and phylogeographic structure of Andes virus in humans and rodents in Chile. Journal of Virology.

[ref-44] Mills JN, Yates TL, Childs JE, Parmenter RR, Ksiazek TG (1995). Guidelines for working with rodents potentially infected with hantavirus. Journal of Mammalogy.

[ref-45] Moreira-Arce D, Vergara PM, Boutin S, Simonetti JA, Briceño C, Acosta-Jamett G (2015). Native forest replacement by exotic plantations triggers changes in prey selection of mesocarnivores. Biological Conservation.

[ref-46] Muñoz-Pedreros A, Murúa R, González L (1990). Nicho ecológico de micromamíferos en un agroecosistema forestal de Chile central. Revista Chilena de Historia Natural.

[ref-47] Murúa AR, González LA, Jofre C (1980). Experimental food preferences of two southern chilean rodents. Journal of Mammalogy.

[ref-48] Murúa R, González LA (1982). Microhabitat selection in two chilean cricetid rodents. Oecologia.

[ref-49] Murúa R, González LA (1986). Regulation of numbers in two Neotropical rodent species in southern Chile. Revista Chilena de Historia Natural.

[ref-50] Murúa R, González LA, Meserve PL (1986). Population ecology of oryzomys longicaudatus philippii (Rondentia: Cricetidae). Journal of Animal Ecology.

[ref-51] Myers N, Mittermeier RA, Mittermeier CG, Fonseca GAB, Kent J (2000). Biodiversity hotspots for conservation priorities. Nature.

[ref-52] Nei M (1982). Evolution of human races at gene level. Human genetics.

[ref-53] Nowakowski AJ, Dewoody JA, Fagan ME, Willoughby JR, Donnelly MA (2015). Mechanistic insights into landscape genetic structure of two tropical amphibians using field-derived resistance surfaces. Molecular Ecology.

[ref-54] Oksanen J, Blanchet G, Friendly M, Kindt R, Legendre P, McGlinn D, Minchin PR, O’Hara RB, Simpson GL, Solymos P, Stevens MHH, Szoecs E, Wagner H (2016). http://cran.r-project.org/package=vegan.

[ref-55] Olson DM (1998). The global 200: a representation approach to conserving the earth’s most biologically valuable ecoregions. Issues in Intermational Conservation.

[ref-56] Ortiz N, Polop FJ, Andreo VC, Provensal MC, Polop JJ, Gardenal CN, González-Ittig RE (2017). Genetic population structure of the long-tailed pygmy rice rat (Rodentia, Cricetidae) at different geographic scales in the Argentinean Patagonia. Journal of Zoology.

[ref-57] Palma RE, Boric-Bargetto D, Torres-Pérez F, Hernández CE, Yates TL (2012a). Glaciation effects on the phylogeographic structure of *Oligoryzomys longicaudatus* (Rodentia : Sigmodontinae) in the Southern Andes. PLOS ONE.

[ref-58] Palma RE, Polop JJ, Owen RD, Mills JN (2012b). Ecology of rodent-associated hantaviruses in the southern cone of South America: Argentina, Chile, Paraguay, and Uruguay. Journal of Wildlife Diseases.

[ref-59] Palma RE, Rivera-Milla E, Salazar-bravo J, Torres-Pérez F, Pardiñas UFJ, Marquet PA, Spotorno AE, Meynard AP, Terry L (2005). Phylogeography of *Oligoryzomys longicaudatus* (Rodentia: Sigmodontinae) in temperate South America. Journal of Mammalogy.

[ref-60] Pesole G, Gissi C, Chirico A De, Saccone C (1999). Nucleotide substitution rate of mammalian mitochondrial genomes. Journal of Molecular Evolution.

[ref-61] Polop FJ, Provensal C, Pini N, Levis SC, Priotto W, Enría D, Calderon G, Costa F, Polop JJ (2010). Temporal and spatial host abundance and prevalence of andes hantavirus in southern Argentina. EcoHeath.

[ref-62] Prugh LR, Hodges KE, Sinclair ARE, Brashares JS (2008). Effect of habitat area and isolation on fragmented animal populations. Proceedings of the National Academy of Sciences of the United States of America.

[ref-63] R Core Team (2016). https://www.r-project.org.

[ref-64] Radespiel U, Bruford MW (2014). Fragmentation genetics of rainforest animals: insights from recent studies. Conservation Genetics.

[ref-65] Ricketts TH (2001). The matrix matters: effective isolation in fragmented landscapes. The American Naturalist.

[ref-66] Rosel PE, France SC, Wang JY, Kocher TD (1999). Genetic structure of harbour porpoise *Phocoena phocoena* populations in the northwest Atlantic based on mitochondrial and nuclear markers. Molecular Ecology.

[ref-67] Saccone C, Pesole G, Sbisà E (1991). The main regulatory region of mammalian mitochondrial DNA: structure-function model and evolutionary pattern. Journal of Molecular Evolution.

[ref-68] Sbisà E, Tanzariello F, Reyes A, Pesole G, Saccone C (1997). Mammalian mitochondrial D-loop region structural analysis: identification of new conserved sequences and their functional and evolutionary implications. Gene.

[ref-69] Secretariat of the Convention on Biological Diversity (2005). Handbook of the convention on biological diversity including its cartagena protocol on biosafety.

[ref-70] Sikes RS, Gannon WL, Mammalogist TAC and UC of the AS (2011). Guidelines of the American society of mammalogists for the use of wild mammals in research. Journal of Mammalogy.

[ref-71] Spotorno OAE, Palma VRE, Valladares FJP (2000). Biología de roedores reservorios de hantavirus en Chile [Biology of rodent reservoirs of hantavirus in Chili]. Revista chilena de infectología.

[ref-72] Taylor AC, Walker FM, Goldingay RL, Ball T, Van Der Ree R (2011). Degree of landscape fragmentation influences genetic isolation among populations of a gliding mammal. PLOS ONE.

[ref-73] Thompson JD, Higgins DG, Gibson TJ (1994). CLUSTAL: improving the sensitivity of progressive multiple sequence alignment through sequence weighting, position-specific gap penalties and weight matrix choice. Nucleic Acids Research.

[ref-74] Toro J, Vega JD, Khan AS, Mills JN, Padula P, Terry W, Yadón Z, Valderrama R, Ellis BA, Pavletic C, Cerda R, Zaki S, Shieh WJ, Meyer R, Tapia M, Mansilla C, Baro M, Vergara A, Concha M, Calderon G, Enria D, Peteres CJ, Ksiazek TG (1998). An outbreak of hantavirus pulmonary syndrome, Chile, 1997. Emerging Infectious Diseases.

[ref-75] Torres-Pérez F, Navarrete-Droguett J, Aldunate R, Yates TL, Mertz GJ, Vial PA, Ferrés M, Marquet PA, Palma RE (2004). Peridomestic Small mammals associated with confirmed cases of human hantavirus disease in southcentral Chile. The American Journal of Tropical Medicine and Hygiene.

[ref-76] Van Etten J (2015). http://cran.r-project.org/package=gdistance.

[ref-77] Van Etten J (2017). R package gdistance: distances and routes on geographical grids. Journal of Statistical Software.

[ref-78] Van Strien MJ, Keller D, Holderegger R (2012). A new analytical approach to landscape genetic modelling: least-cost transect analysis and linear mixed models. Molecular Ecology.

[ref-79] Vigilant L, Stoneking M, Harpending H, Hawkes K, Wilson AC (1991). African populations and the evolution of human mitochondrial DNA. Science.

[ref-80] Vos CC, Stumpel AHP (1995). Comparison of habitat-isolation parameters in relation to fragmented distribution patterns in the tree frog (*Hyla arborea*). Landscape Ecology.

[ref-81] Wakeley J (1993). Substitution rate variation among sites in hypervariable region 1 of human mitochondrial DNA. Journal of Molecular Evolution.

[ref-82] Walker R, Craighead L (1997). Analyzing wildlife movement corridors in Montana using GIS.

[ref-83] Wilcove DS, McLellan CH, Dobson AP (1986). Habitat fragmentation in the temperate zone. Conservation biology: the science of scarcity and diversity.

[ref-84] Willi Y, Van Buskirk J, Schmid B, Fischer M (2007). Genetic isolation of fragmented populations is exacerbated by drift and selection. Journal of Evolutionary Biology.

[ref-85] Young A, Boyle T, Brown T (1996). The population genetic consequences of habitat fragmentation for plants. Tree.

