# Supplementary material for: Does silvoagropecuary landscape fragmentation affect the genetic diversity of the sigmodontine rodent Oligoryzomys longicaudatus?
Source: PeerJ. 2017 Sep 29;5:e3842. doi: 10.7717/peerj.3842 (PMC5624292; doi:10.7717/peerj.3842)
Supplement: Table S1 — Criteria for delimitation of patch areas. Criteria were designed visualizing the patches in Google Earth Pro v 7.1 (http://www.google.com/earth/) and based on Soil Use Cover data of the Chilean National Environmental Information System (http://ide.mma.gob.cl/). [file peerj-05-3842-s001.pdf]

Criteria for delimitation of patch areas.

Patch	Criteria*	Matrix
FR1	Delimited by forestry paths at its upper, left and bottom, and change of vegetation.	Plantation forestry Grassland and shrubland
FR2	Delimited by change of vegetation.	Plantation forestry
FR3	Delimited by forestry paths at its left and right sides, and change of vegetation.	Plantation forestry
FR4	Delimited by forestry paths at all its sides.	Plantation forestry
FR5	Delimited by forestry paths at its upper, left and right sides, and change of vegetation.	Plantation forestry Grassland and shrubland

*Criteria were designed visualizing the patches in Google Earth Pro v 7.1 (http://www.google.com/earth/) and based on Soil Use Cover data of the Chilean National Environmental Information System (http://ide.mma.gob.cl/).
