# Supplementary material for: Does silvoagropecuary landscape fragmentation affect the genetic diversity of the sigmodontine rodent Oligoryzomys longicaudatus?
Source: PeerJ. 2017 Sep 29;5:e3842. doi: 10.7717/peerj.3842 (PMC5624292; doi:10.7717/peerj.3842)
Supplement: Table S2 — Sampling site, patch name, coordinates of specimens, GenBank accession number and voucher number of each specimen used in this study. [file peerj-05-3842-s002.pdf]

Sampling site, patch name, coordinates of specimens, Genbank accession number and voucher number of each specimen used in this study.

Patch	Latitude	Longitude	Accession	Voucher
FR1	-39.4920278	-72.5456111	KY211763	NK142648
FR1	-39.4920278	-72.5456111	KY211764	NK142818
FR1	-39.4920278	-72.5456111	KY211765	NK142824
FR1	-39.4920278	-72.5456111	KY211766	NK142830
FR1	-39.4920278	-72.5456111	KY211783	NK142630
FR1	-39.4920278	-72.5456111	KY211784	NK142631
FR1	-39.4920278	-72.5456111	KY211785	NK142635
FR1	-39.4920278	-72.5456111	KY211786	NK142636
FR1	-39.4920278	-72.5456111	KY211787	NK142645
FR1	-39.4920278	-72.5456111	KY211788	NK142813
FR1	-39.4920278	-72.5456111	KY211799	NK142637
FR1	-39.4920278	-72.5456111	KY211800	NK142629
FR2	-39.4902778	-72.5465833	KY211801	NK142876
FR2	-39.4902778	-72.5465833	KY211802	NK142855
FR2	-39.4902778	-72.5465833	KY211803	NK142859
FR2	-39.4902778	-72.5465833	KY211804	NK142874
FR2	-39.4902778	-72.5465833	KY211805	NK142863
FR2	-39.4902778	-72.5465833	KY211806	NK142867
FR2	-39.4902778	-72.5465833	KY211807	NK142838
FR2	-39.4902778	-72.5465833	KY211808	NK142848
FR2	-39.4902778	-72.5465833	KY211809	NK142851
FR2	-39.4902778	-72.5465833	KY211810	NK142853
FR2	-39.4902778	-72.5465833	KY211811	NK142860
FR2	-39.4902778	-72.5465833	KY211812	NK142865

FR2	-39.4902778	-72.5465833	KY211813	NK142875
FR2	-39.4902778	-72.5465833	KY211814	NK142880
FR2	-39.4902778	-72.5465833	KY211815	NK142885
FR2	-39.4902778	-72.5465833	KY211816	NK142887
FR2	-39.4902778	-72.5465833	KY211817	NK142883
FR2	-39.4902778	-72.5465833	KY211818	NK142886
FR3	-39.4862222	-72.5290278	KY211820	NK142667
FR3	-39.4862222	-72.5290278	KY211821	NK142668
FR3	-39.4862222	-72.5290278	KY211822	NK142947
FR3	-39.4862222	-72.5290278	KY211823	NK142659
FR3	-39.4862222	-72.5290278	KY211824	NK142660
FR3	-39.4862222	-72.5290278	KY211825	NK142677
FR3	-39.4862222	-72.5290278	KY211826	NK142955
FR3	-39.4862222	-72.5290278	KY211827	NK142943
FR3	-39.4862222	-72.5290278	KY211828	NK142957
FR4	-39.4878889	-72.5265278	KY211829	NK142690
FR4	-39.4878889	-72.5265278	KY211830	NK142691
FR4	-39.4878889	-72.5265278	KY211831	NK142694
FR4	-39.4878889	-72.5265278	KY211832	NK142703
FR4	-39.4878889	-72.5265278	KY211833	NK142710
FR4	-39.4878889	-72.5265278	KY211834	NK142685
FR4	-39.4878889	-72.5265278	KY211835	NK142687
FR4	-39.4878889	-72.5265278	KY211836	NK142702
FR4	-39.4878889	-72.5265278	KY211837	NK142704
FR4	-39.4878889	-72.5265278	KY211838	NK160034
FR4	-39.4878889	-72.5265278	KY211839	NK142682
FR4	-39.4878889	-72.5265278	KY211840	NK142688

FR4	-39.4878889	-72.5265278	KY211841	NK142689
FR4	-39.4878889	-72.5265278	KY211842	NK142692
FR4	-39.4878889	-72.5265278	KY211843	NK142698
FR4	-39.4878889	-72.5265278	KY211844	NK142700
FR4	-39.4878889	-72.5265278	KY211845	NK142701
FR4	-39.4878889	-72.5265278	KY211846	NK142705
FR4	-39.4878889	-72.5265278	KY211847	NK142707
FR4	-39.4878889	-72.5265278	KY211848	NK142708
FR4	-39.4878889	-72.5265278	KY211849	NK142709
FR4	-39.4878889	-72.5265278	KY211850	NK142716
FR4	-39.4878889	-72.5265278	KY211851	NK142719
FR4	-39.4878889	-72.5265278	KY211852	NK160047
FR5	-39.4909167	-72.5390278	KY211853	NK142731
FR5	-39.4909167	-72.5390278	KY211854	NK142741
FR5	-39.4909167	-72.5390278	KY211855	NK142754
FR5	-39.4909167	-72.5390278	KY211856	NK142722
FR5	-39.4909167	-72.5390278	KY211857	NK142725
FR5	-39.4909167	-72.5390278	KY211858	NK142726
FR5	-39.4909167	-72.5390278	KY211859	NK142727
FR5	-39.4909167	-72.5390278	KY211860	NK142736
FR5	-39.4909167	-72.5390278	KY211861	NK142760
FR5	-39.4909167	-72.5390278	KY211862	NK142764
